# Development and Validation of the Computerized Family Relations Test for Children

**DOI:** 10.3389/fpsyg.2015.01687

**Published:** 2015-11-12

**Authors:** Ilona Skoczeń, Jan Cieciuch, Johan H. L. Oud, Kai Welzen

**Affiliations:** ^1^The Institute of Psychology, Cardinal Stefan Wyszyński University, Warsaw, Poland; ^2^Behavioural Science Institute, Radboud University, Nijmegen, Netherlands

**Keywords:** assessment, family relations, children, CFRT, computer

## Abstract

The aim of the present study was to develop and investigate the psychometric properties of the Computerized Family Relations Test (CFRT) for children. This test assesses the quality of family relationships with the mother and father from a child’s perspective. The CFRT consists of six scales relating to control (Restrictiveness and Justice), and support (Affection, Vulnerability, Acknowledgment, and Trust) within the family relationships. CFRT is an innovative approach to the Dutch Nijmegen Family Relations Test (NFRT) developed by Oud and Welzen (1989). The administration of the test has been computerized and graphical representations of female and male silhouettes were included to facilitate the child’s parental identification. In total, 404 primary school children, aged 8 to 13 years (*M* = 11.0; *SD* = 1.17), took part in this study. The CFRT’s reliability was assessed by McDonald’s omega coefficients, and ranged from 0.71 to 0.86, except for Vulnerability which achieved the lowest reliability 0.57 for mothers’ ratings and 0.56 for fathers’ ratings. The test–retest procedure revealed higher stability for the ratings on father-child relationships of 0.71 compared to mother-child relationships of 0.67. Confirmatory factor analysis indicated that a six-factor model provided an adequate fit. Measurement invariance across the children’s assessments of the quality of family relationships was achieved. The construct validity of CFRT was assessed by examining differences in the child’s ratings of the relationships with the mother and father, the child’s gender, and associations of CFRT scales with other variables such as depression, anxiety symptoms, and prosocial behavior.

## Introduction

Prior studies in family psychology have indicated the need to highlight children’s perspectives on family relationships in research and practice, as children are very careful observers who can provide distinctive views of overall family functioning, parenting and the quality of interpersonal relationships (e.g., Milkie et al., 1997). Researchers agree that special emphasis should be placed on the quality of measures that aim to obtain data on the quality of family relationships directly from children. These instruments must be adjusted to the child’s current developmental stage, use age-appropriate, understandable language, and have an engaging design to hold the child’s attention, to ensure the highest measurement accuracy (Strachan et al., 2010). Traditional pen and paper questionnaires might be difficult to complete, especially for young children, because they require good reading and attention skills. Research on testing technology has shown that children prefer computer-based testing (Sim and Horton, 2005). Such testing impacts scores positively, for example, in the case when only one item is displayed at a time on the computer screen, and leads to greater focus and closure (Clariana and Wallace, 2002). Furthermore, valuable information can be obtained in a short period of time.

Despite the importance of this topic, there has been a measurement gap in analyzing family relations from the child’s perspective in both research and practice. Very few instruments have been developed to elicit children’s feelings and perceptions of family relationships (Strachan et al., 2010). The most widely used measure, The Family Relations Test (FRT), was developed by Anthony and Bene (1957) nearly 60 years ago and continues to encounter problems with standardized scoring, administration, question wording, and use with non-white ethnic groups (Parkin, 2001). The Structured Child Assessment of Relationships in Families (SCARF; Strachan et al., 2010) tackles important domains, such as emotional security, and positive and negative parenting; however, the child is restricted to selecting only one family member when answering a question (e.g., “Who gives you a treat or something special when you are good?”). The Child–Parent Relationship Test (ChiP–C; Titze et al., 2014) is clinically oriented and contains domains that relate to resources and risks; however, ChiP–C is sensitive to cultural differences and, therefore, requires further validation. The Network of Relationships Inventory (NRI; Furman and Buhrmester, 1985) has been used to assess a wide range of qualities of relationships with parents, siblings, grandparents, friends, and teachers, in which participants use the same set of items to describe their relationship with each of several members in their social network. Several attempts have been made to develop attachment styles measures in the form of narratives, such as the MacArthur Story Stem Battery (MSSB; Bretherton et al., 2003), the Attachment Story Completion Task (ASCT; Bretherton et al., 1990), and the Manchester Child Attachment Story Task (MCAST; Green et al., 2000), in which participants are asked to continue introduced attachment-relevant story stems. Although children find playing with the dolls engaging, these measures have been criticized because task administration requires good attention and control skills. Moreover, children need to focus on the technique, follow the researcher’s or clinician’s guidelines, and express their own views about the family simultaneously (Poehlmann et al., 2014). In addition, these instruments are time consuming and expensive to administer, as mostly they require prior user training and the purchase of appropriate equipment.

### Computerized Family Relations Test and its Origins

The Computerized Family Relations Test (CFRT) for children is an innovative measure that aims to assess the quality of family relationships from the child’s perspective. However, the CFRT has its origins in the Dutch Nijmegen Family Relations Test (NFRT; Oud and Welzen, 1989) that has been applied in several studies (e.g., Mathijssen et al., 1998; Delsing et al., 2003, 2005a,b).

During the development of the NFRT, Oud and Welzen (1989) attempted to operationalize family theories in psychological research, resulting in the development of a family relationships model based on the following six dimensions: Restrictiveness, Affection, Vulnerability, Justice, Acknowledgment, and Trust. The model is grounded in the theoretical framework of two systemic family therapists, Helm Stierlin’s (1978) binding theory and Ivan Boszormenyi-Nagy’s (Boszormenyi-Nagy and Spark, 1984) loyalty theory, various experiences of family and child psychotherapists, and information gathered directly from children. Two dimensions – Restrictiveness and Affection – originate from the psychoanalytically-oriented binding theory of Stierlin (1978), which refers to different types of transactions between the parent and the child on the id, ego and superego levels. The remaining four dimensions–Justice, Vulnerability, Acknowledgment and Trust–form key elements of the loyalty theory of Boszormenyi-Nagy (Boszormenyi-Nagy and Spark, 1984), which assumes that interpersonal perceptions of loyalty within the family are the product of the closely intertwined but distinctive dimensions of justice and trust. On one hand, children perceive their parents as just if they feel they are being treated fairly in the context of family obligations. On the other hand, children perceive their parents as trustworthy if they feel valuable and loved.

Based on this model, Fitriana (2011) developed an Indonesian version of the NFRT called the Bandung Family Relations Test (BFRT). The confirmatory factor analysis showed that the six dimensions of family relationships could be divided into two second-order factors, which describe *control* (Restrictiveness and Justice), and *support* (Affection, Vulnerability, Acknowledgment, and Trust). The division of control versus support is a common categorization in research on parent–child relationships (Tynkkynen et al., 2012; Hooghe et al., 2013).

The CFRT consists of 67 items, the same as the original NFRT, forming six scales: Restrictiveness (12 items e.g., “This person often bosses me around”), Affection (10 items e.g., “If I go away, this person will really miss me”), Vulnerability (7 items e.g., “I like to know what this person thinks or feels”), Justice (12 items e.g., “If I promise this person something, then I also do it”), Acknowledgment (13 items e.g., “This person often tells me that I do something well”), and Trust (13 items e.g., “This person protects me”). The main change is in the administration of the CFRT from a traditional pen-and paper questionnaire to a computerized version. While developing the CFRT, we translated the items in accordance with the International Test Commission (ITC) guidelines for translating and adapting tests in cross-cultural research (Brislin, 1986; Hambleton, 2005). The procedure included the following steps: (1) forward-translation of all items from the existing English version of the NFRT to Polish, (2) consultation over the results with two experts in child psychology and cross-cultural research regarding the linguistic, developmental and cultural suitability of the test, (3) back-translation of all items from Polish to English, (4) receiving authors’ comments and suggestions, (5) preparation of the final version of CFRT prior to the introduction of all recommended modifications.

**FIGURE 1:**
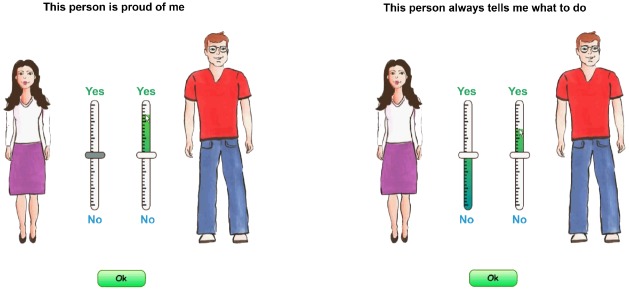
**Screen prints of the CFRT**.

The CFRT has been programmed in Flash software and consists of an instruction, an animated guide on how to answer the questions, and a set of exemplar pictures of female and male silhouettes, from which the test-taker chooses those most similar to his or her mother and father. The graphical representations of parents facilitate the child’s parental identification, especially among younger children. Children assess relationships with their father and mother separately, with the possibility of selecting a single parent option. The questions appear in two synchronized ways, displaying at the top of the screen above the silhouettes and read aloud to the test-taker by a previously recorded voice. In contrast to the NFRT and BFRT, CFRT has a continuous response scale, which we believe is more accurate than traditional Likert-type scales and does not limit the test-taker to one particular category. With the use of a specially designed slider bar that is similar to a thermometer, the child is asked to indicate the extent to which he or she agrees that the item is applicable to each parent, ranking from totally agree (top scale—Yes) to totally disagree (bottom scale—No), or uncertain (middle point), as presented in Figure 1. Data collected by this approach meet the assumptions of many statistical analyses, including confirmatory factor analysis—CFA (Treiblmaier and Filzmoser, 2011).

We recorded and randomized the display of the items to minimize the impact of reading ability on participants’ responses (Borelli et al., 2010) and to avoid order effect, decreases in children’s motivation (e.g., when seeing that others perform faster), and increases in carry-over, fatigue, priming, and learning effects. It is worth noting that item order differentiation has become a common practice in psychological research (Khorramdel and Frebort, 2011).

## The Current Study

The aim of this study is to report the development and psychometric properties of the CFRT. We intend to confirm the following seven assumptions: (1) the reliability of the CFRT will be acceptable and comparable to the original Dutch NFRT; (2) the construct stability will be satisfactory; (3) the six-factor CFA model will fit the data; (4) the measurement of family relationships across the mother and the father ratings will be supported; regarding the construct validity we expected (5) perceptions of the family relationships with the mother and the father to differ, such that the ratings of child-mother relationships will be higher on *support*, whereas the ratings of child-father relationships will be higher on the *control* dimension (as found in Oud and Welzen, 1989; Fitriana, 2011); (6) gender differences will occur in the ratings of family relationships with both parents. This assumption is in line with previous research that showed parents relate to their sons and daughters differently (e.g., Gurwitz and Dodge, 1975) and use different parenting techniques (Chao, 2011); (7) significant associations between the CFRT dimensions and other psychological variables will be observed. *Control* within family relationships is expected to correlate positively with the child’s depression and anxiety symptoms, whereas *support* is assumed to correlate negatively, as found in previous studies (e.g., Cole and McPherson, 1993; Kim et al., 2008; Creveling et al., 2010). Justice in family relations is expected to correlate positively with the child’s prosocial behavior, as found in Dunn et al. (2001).

## Materials and Methods

### Participants

In total, 404 Polish children, ranging in age from 8 to 13 years (*M* = 11.0; *SD* = 1.17), participated in this study. Of the participating children, 54% were girls (*N* = 219) and 46% boys (*N* = 185). All participants were primary school pupils in grades three to six.

Additionally, a randomly selected group of the children *N* = 60 (55% girls and 45% boys), aged 8–13 years (*M* = 11.0; *SD* = 1.16), participated in the test–retest procedure after a 6-week interval.

### Procedure and Measures

First, the institutional review board at the Psychology Institute, Cardinal Stefan Wyszyński University in Warsaw reviewed this project and gave us permission to implement it. An invitation letter to take part in a research project on the role of family relationships in childhood and adolescence was sent to 12 public primary schools across Poland, of which ten agreed to participate. After we gained consent from the school principals to carry out the project, the main researcher attended parent–teacher meetings that took place regularly at the schools to introduce the nature of the project and invite parents and their children to participate. Parents received a study description with a consent form for their children to participate in this study. Of all invited parents, 87% provided written consent for their children to participate. Only children with parental written consent participated. Participation in this study was voluntary and anonymous. The research was conducted at schools during Information Technology (IT) lessons and in groups of 10 to 12 children with the presence of one researcher and one trained graduate student to ensure the standardized setting of the data collection and participants’ confidentiality. The results were analyzed at the group level and for scientific purposes exclusively.

The research equipment contained a computer, with a headset. Before the study began, all participating children were asked for oral permission to take part. The main researcher, with the help of a trained graduate student, explained the nature of the study and its procedure. Children were also informed about anonymity and their right to withdraw from the testing at any time without consequences. The researchers stressed that there were no good or bad answers and children were asked to provide honest answers, reflecting their perceptions about the quality of relationships in their families. The first item was neutral to enable the children to practice answering the questions. The researchers were available during the whole study to provide support in case of any questions or difficulties children might have had.

#### Depression and Anxiety Symptoms

Revised Child Anxiety and Depression Scale (RCADS; Chorpita et al., 2000) is a 47-item measure of depression and anxiety symptoms in children that consists of two general scales, Anxiety (α = 0.75) and Depression (α = 0.77). Items are rated on a four-point Likert scale (0 = *never* to 3 = *always*).

#### Prosocial Behavior

Strengths and Difficulties Questionnaire (SDQ; Goodman, 1997) is a 25-item screening instrument that measures children’s strengths and difficulties in five domains: Emotional Symptoms, Conduct Problems, Hyperactivity/Inattention, Peer Relationships Problems, and Prosocial Behavior. For the purpose of the current study, only the Prosocial Behavior (α = 0.68) scale was used. Answers were rated on a three-point Likert scale (0 = *not true at all* to 2 = *definitely true*).

## Analysis

All reliability and validity analyses were performed with SPSS version 21.0 (IBM Corp., 2012). We tested the reliability of CFRT through the assessment of internal consistency with the use McDonald’s omega (McDonald, 1999) and test–retest coefficients. Confirmatory factor analysis and measurement invariance across the mother and father ratings were performed in statistical software for structural equation modeling—using AMOS version 21.0 (Arbuckle, 2011). Although previous research showed that analyses based on individual items or item parcels are equally appropriate (Hau and Marsh, 2004), parceling is recommended when a scale contains more than five items (Bagozzi and Heatherton, 1994) to increase the reliability of responses, obtain more stable parameter estimates, and simplify model interpretation (Bandalos and Finney, 2001). The CFRT scales consist of seven to thirteen items; therefore, the parceling approach was applied for the purpose of this study. Items from each scale were grouped randomly into three parcels and each parcel contained three to five items. We tested measurement invariance to assess whether the same construct was being measured across mother and father ratings. Furthermore, we tested construct validity by examining differences in the child’s ratings of the relationships with the mother and the father, the child’s gender, and the associations of CFRT scales with other variables such as depression, anxiety symptoms, and prosocial behavior.

## Results

### Reliability

We examined the reliability of the CFRT scales by calculating McDonald’s omega coefficients for each scale, separately for mother and father ratings, and the internal stability was assessed through a test–retest procedure after a 6-week interval. Reliability estimates are presented in Table 1.

**TABLE 1:** **Model-based scale score reliabilities with Mcdonald’s omega (Ω) with bootstrapped 95% confidence intervals of the CFRT and test–retest coefficients**.

**Scale**	**Mother *N* = 401**		**Father *N* = 395**	
	**ω (95% CI)**	**test–retest *r***	**ω (95% CI)**	**test–retest *r***	
Restrictiveness	0.82 (0.79–0.84)	0.71	0.82 (0.79–0.84)	0.72
Affection	0.76 (0.72–0.80)	0.68	0.81 (0.77–0.84)	0.74
Vulnerability	0.57 (0.49–0.64)	0.67	0.56 (0.47–0.63)	0.71
Justice	0.71 (0.65–0.77)	0.68	0.74 (0.67–0.78)	0.69
Acknowledgment	0.84 (0.80–0.87)	0.62	0.86 (0.83–0.89)	0.66
Trust	0.84 (0.81–0.88)	0.67	0.86 (0.83–0.89)	0.74

With regard to mother and father ratings, CFRT showed good reliabilities for all scales, with Acknowledgment and Trust scoring highest, ω = 0.84 for mothers and ω = 0.86 for fathers, respectively. Parallel to the Dutch data, in the Polish results, Vulnerability achieved the lowest reliability, ω = 0.57 for mothers and ω = 0.56 for fathers. Test–retest coefficients showed higher stability for father ratings *r* = 0.71 than for mother ratings *r* = 0.67.

### Factorial Structure of the CFRT

**FIGURE 2:**
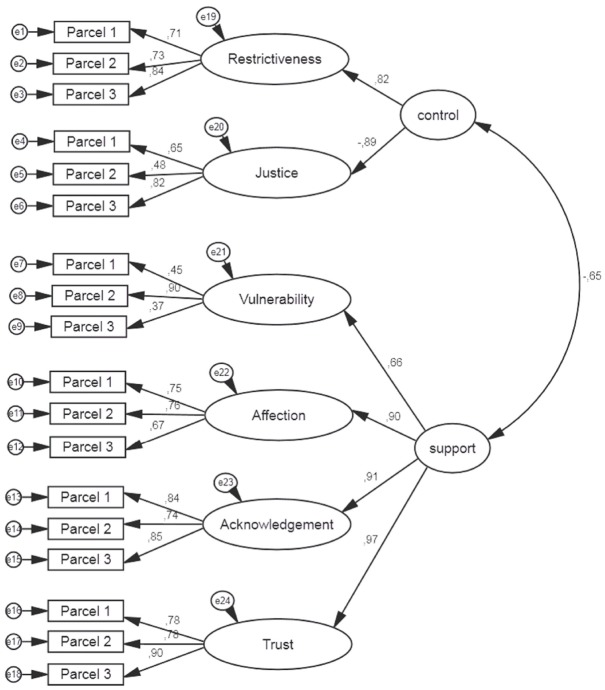
**CFA model for the child-mother dyad in the CFRT**.

**FIGURE 3:**
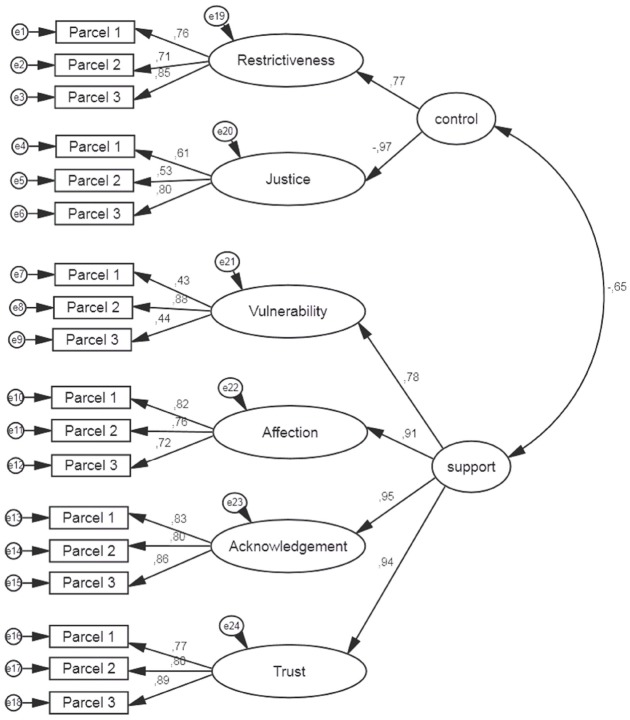
**CFA model for the child-father dyad in the CFRT**.

We tested two models, first-order CFA and second-order CFA, separately for mothers and fathers. The first-order CFA model consisted of six latent variables. Each latent variable was built upon three parcels as observed variables. The CFRT’s factor structure was examined using chi-squared, the standardized root mean square residual (SRMR), the comparative fit index (CFI), and the root mean square error of approximation (RMSEA). A non-significant chi-squared, SRMR values below 0.08, CFI values above 0.95, and RMSEA values below 0.06 are recommended (Hu and Bentler, 1999). Model fit coefficients (as presented in Table 2) were acceptable; thus, it can be concluded that the measurement model of six separate dimensions fit the data well. However, some sets of dimensions were highly intercorrelated, which might indicate that children did not differentiate between them. In the CFA for mothers, three correlations were above 0.80 (the highest correlations were between Acknowledgment–Trust and Affection–Trust and equaled 0.88). In the CFA for fathers, four correlations were above 0.80 (the highest was between Acknowledgment–Trust and equaled 0.90). Thus, based on previous research assumptions (Fitriana, 2011; Hooghe et al., 2013), we included *control* and *support* in the second-order CFA model. Model fit coefficients for the two types of models tested are presented in Table 2. Second-order models are presented graphically in Figure 2 for mothers and in Figure 3 for fathers.

**TABLE 2:** **Model fit of the six scale CFRT in CFA**.

**Model**	***χ*^2^**	**df**	**CFI**	**RMSEA**	**SRMR**
Mother First order CFA	442.2	120	0.910	0.082 (0.074–0.090)	0.079
Second order CFA	486.6	128	0.900	0.084 (0.076–0.092)	0.085
Father First order CFA	411.1	120	0.925	0.078 (0.070–0.086)	0.072
Second order CFA	449.1	128	0.918	0.079 (0.071–0.087)	0.078

CFI, comparative fit index; RMSEA, root mean square error of approximation; SRMR, standardized root mean square residual.

The analysis confirmed our expectations. The appropriate parcels loaded onto the six latent variables. Additionally, six scales loaded onto two second-order factors in the second-order CFA. Restrictiveness and Justice loaded onto the *control* factor, and Vulnerability, Affection, Acknowledgment, and Trust loaded onto the *support* factor.

### Measurement Invariance

To test whether CFRT measures the same construct, i.e., family relationships, in the same manner across the child’s parents, measurement invariance across the children’s assessments of the quality of family relations with mother and father was tested (the results are presented in Table 3).

**TABLE 3:** **Fit indices for measurement invariance models**.

**Model**	***χ*^2^**	**Df**	**CFI**	**RMSEA**	**SRMR**
Configural	779.8	264	0.923	0.050 (0.046–0.054)	0.093
Metric at the first order part	794.7	276	0.922	0.049 (0.045–0.053)	0.093
Scalar at the first order part	824.8	288	0.920	0.048 (0.045–0.052)	0.094
Structural weights (equality of the loading at the second order part)	834.4	292	0.919	0.048 (0.045–0.052)	0.094

CFI, comparative fit index; RMSEA, root mean square error of approximation, SRMR, standardized root mean square residual.

The following levels of measurement invariance were tested: the configural level (all conditions have the same pattern of factor loadings); the metric level (factor loadings are constrained to be equal across the compared conditions); and the scalar level (the indicator intercepts are constrained to be equal across various conditions; Vandenberg and Lance, 2000; Davidov et al., 2014). These three levels were examined in the first-order CFA model. In addition, we tested whether the second-order factors had the same meaning in the assessment of relations with mothers and fathers by constraining the loadings in the CFA to be equal. The results showed that changes in CFI were less than 0.01, changes in RMSEA were less than 0.015, and changes in SRMR were less than 0.03, which supports invariance of the measurement across mothers and fathers, according to Chen (2007).

### Construct Validity

Repeated-measures MANOVA was conducted to test gender effect on the perception of family relations with mother versus father ratings within factor and child’s gender between factor. The results showed significant multivariate effects for five out of the six CFRT dimensions: Affection *F*_(1,390)_ = 54.37, *p* < 0.001; Vulnerability *F*_(1,390)_ = 46.86, *p* < 0.001; Justice *F*_(1,392)_ = 5.05, *p* < 0.05; Acknowledgment *F*_(1,390)_ = 23.73, *p* < 0.001; and Trust *F*_(1,390)_ = 7.94, *p* < 0.01 with mother receiving higher ratings compared to father ratings. The multivariate effect for Restrictiveness was not significant *F*_(1,390)_ = 1.60, *p* = 0.206. No significant interaction effects between mother-father ratings and the child’s gender were observed.

All scales of the CFRT were expected to be associated with measures of psychological adjustment. For all assumptions made, Pearson’s bivariate correlations were used to determine the associations between the CFRT scales and target variables, including depression, anxiety symptoms, and prosocial behavior measured with the RCADS and SDQ. The findings revealed that higher ratings on Restrictiveness were associated with higher levels of child’s depressive symptoms in relations with their mother (*r* = 0.38; *p* < 0.01) and father (*r* = 0.31; *p* < 0.01) and with higher levels of children’s anxiety (mother *r* = 0.34; *p* < 0.01 and father *r* = 0.35; *p* < 0.01). Justice in relations with both parents was negatively correlated with depression (mother ratings *r* = –0.45; *p* < 0.01 and father ratings *r* = –0.40; *p* < 0.01) and anxiety (mother ratings *r* = –0.34; *p* < 0. 01 and father ratings *r* = –0.38; *p* < 0.01). Three of the four *support* factors (Affection, Acknowledgment, Trust) negatively correlated with depression. Justice correlated positively with prosocial behavior (mother ratings *r* = 0.31; *p* < 0.01 and father ratings *r* = 0.33; *p* < 0.01).

## Discussion

The current study aimed to address an existing gap in the assessment of family functioning from the child’s perspective by introducing a reliable and developmentally appropriate measure of family relationships for children, administered on computer. This paper focused on the development of a computerized measure of family relationships in middle childhood—the CFRT.

The current findings revealed the reliability and validity of the CFRT scales. Reliability was supported by internal consistency and test–retest reliability. However, Vulnerability achieved the lowest reliability coefficients, which might be caused by several factors. According to Oud and Welzen (1989), Vulnerability refers to the degree to which a child is able to sense the signs of pain and sorrow experienced by parents. It also creates the basis of sympathy and empathy in human relations. High scores on this dimension imply that the child and parent have much concern for each other. The low reliability coefficients might be due to the relatively high multidimensionality of items and the lowest number of items (seven) when compared to the remaining five CFRT scales.

Furthermore, we introduced the confirmatory factor analytic approach to test the factorial validity of the CFRT. The results obtained from the CFA indicated that the six-factor model appeared to provide an adequate fit. However, correlations between four scales reached a high value (above 0.9) which was not acceptable, therefore, the six CFRT dimensions are recommended to be divided into two groups: *control* and *support*, which we tested in the second-order CFA model, as suggested by Fitriana (2011). We achieved measurement variance, which means that the same construct was measured across mother and father ratings.

Overall, mothers achieved higher ratings on all four support dimensions and one control dimension (Justice) which partly supports our hypothesis. These results with mother ratings higher in support are in line with Oud and Welzen’s (1989) study and previous research that found mothers express more empathy in family interactions than fathers (e.g., Chao, 2011). In contrast to our hypothesis, we found mothers received higher ratings on Justice compared to father ratings. This finding might be due to parental role models having shifted over the past several decades with mothers taking on a more authoritarian role and fathers getting more involved in family life and forming more affection-based relationships with their children (Aldous, 1998; Delsing et al., 2003; Buswell et al., 2012) rather than being mainly responsible for maintaining discipline. Research on the importance of fathers’ engagement in family life and their contributions to child development has increased recently (Dette-Hagenmeyer et al., 2014). We found no differences between mother and father ratings in the Restrictiveness dimension. In contrast to our hypothesis, we found no significant interaction effects between mother-father ratings and the child’s gender.

Furthermore, we tested associations of CRFT with measures of psychological adjustment—depression, anxiety symptoms, and prosocial behavior. In accordance with our hypothesis, we found a strong association between high ratings on Restrictiveness in the relationships with both parents and child’s depression and anxiety symptoms. The other scale from the control dimension—Justice—was positively related to prosocial behavior (e.g., helping others). Children who are treated in a fair way perceive the world as just and are more willing to help others. Higher levels of support—Affection, Acknowledgment and Trust—were related to lower levels of child depression, which also supported our hypothesis. In contrast to our expectations, Vulnerability ratings were positively related to the child’s depression. This finding also contrasts with a previous study of Kim et al. (2008), who found that low levels of family support influence greater levels of depressive symptoms in children and adolescents. A possible explanation could lie in children’s level of empathy, as those who more likely to observe signs of sorrow in their parents are, in general, more emotional and sensitive and, thus, more prone to develop depressive symptoms. According to Oud and Welzen (1989), Vulnerability is supposed to create the basis of sympathy and empathy in human relations; however, high ratings on this dimension imply that the child and parent have much concern for each other. Therefore, the levels of mutual worry might be so high that they lead to the emergence of depressive symptoms in the child as a result.

To summarize, our data provided evidence for the psychometric properties of the CFRT. We found the computer technique to be engaging and enabled children to express their feelings regarding the quality of family relationships accurately, in a non-verbal way. Children find the whole assessment process enjoyable and it is relatively short to administer, on average 20 min. To our knowledge, the current study is the first to adapt a computerized assessment tool to study family relationships from the child’s perspective in this age group. Although the software was programmed in the Polish language and aimed at Caucasian participants, other linguistic and context-appropriate versions can be prepared. The CFRT requires minimal training for administration and can be performed on any standard PC or a laptop, making it a valuable assessment tool for both research and practice.

### Conflict of Interest Statement

The authors declare that the research was conducted in the absence of any commercial or financial relationships that could be construed as a potential conflict of interest.

## References

[B1] Aldous J. (1998). “The changing concept of fatherhood,” in The Family, Contemporary Perspectives and Challenges, ed. Matthijs K. (Leuven: Leuven University Press), 4–19.

[B2] Anthony E. J. Bene E. (1957). A technique for the objective assessment of the child’s family relations. J. Ment. Sci. 103, 541–555. 13449562 10.1192/bjp.103.432.541

[B3] Arbuckle J. (2011). IBM SPSS AMOS 20 User’s Guide. *Amos Dev. Cooperation* Available at: ftp://public.dhe.ibm.com/software/analytics/spss/documentation/amos/20.0/en/Manuals/IBM_SPSS_Amos_User_Guide.pdf

[B4] Bagozzi R. P. Heatherton T. F. (1994). A general approach to representing multifaceted personality constructs: application to state self-esteem. Struct. Equ. Modeling 1, 35–67. 10.1080/10705519409539961

[B5] Bandalos D. L. Finney S. J. (2001). “Item parceling issues in structural equation modeling,” in Advanced Structural Equation Modeling: New Developments and Techniques, eds Marcoulides G. A. S. Randall E. (Mahwah, NJ: Lawrence Erlbaum Associates), 269–296.

[B6] Boszormenyi-Nagy I. Spark G. M. (1984). Invisible Loyalties: Reciprocity in Intergenerational Family Therapy. New York: Brunner & Mazel.

[B7] Borelli J. L. David D. H. Crowley M. J. Mayes L. C. (2010). Links between disorganized attachment classification and clinical symptoms in school-aged children. J. Child Fam. Stud. 19, 243–256. 10.1007/s10826-009-9292-8

[B8] Bretherton I. Oppenheim D. Emde R. N. the MacArthur Narrative Working Group (2003). “The MacArthur Story Stem Battery,” in Revealing the Inner Worlds of Young Children: The MacArthur Story Stem Battery and Parent—Child Narratives, eds Emde R. N. Wolf D. P. Oppenheim D. (New York: Oxford University Press), 55–80.

[B9] Bretherton I. Ridgeway D. Cassidy J. (1990). “Assessing internal working models of the attachment relationship: an Attachment Story Completion Task for 3-year-olds,” in Attachment in the Preschool Years: Theory, Research, and Intervention, eds Greenberg M. T. Cicchetti D. Cummings E. M. (Chicago: University of Chicago Press), 273–308.

[B10] Brislin R. W. (1986). “The wording and translation of research instruments,” in Cross-cultural research methodology series: Field methods in cross cultural research, eds Lonneri W. J. Berry J. W. (Beverly Hills, CA: Sage), 137–164.

[B11] Buswell L. Zabriskie R. B. Lundberg N. Hawkins A. J. (2012). The relationship between father involvement in family leisure and family functioning: the importance of daily family leisure. Leis. Sci. 34, 172–190. 10.1080/01490400.2012.652510

[B12] Chao M. R. (2011). Family interaction relationship types and differences in parent–child interactions. Soc. Behav. Personal. 39, 897–914. 10.2224/sbp.2011.39.7.897

[B13] Chen F. F. (2007). Sensitivity of goodness of fit indexes to lack of measurement invariance. Struct. Equ. Modeling 14, 464–504.

[B14] Chorpita B. F. Yim L. Moffitt C. E. Umemoto L. A. Francis S. E. (2000). Assessment of symptoms of DSM IV anxiety and depression in children: a revised child anxiety and depression scale. Behav. Res. Ther. 38, 835–855. 10.1016/S0005-7967(99)00130-8 10937431

[B15] Clariana R. Wallace P. (2002). Paper-based vs. computer-based assessment: key factors associated with the test mode effect. Br. J. Educ. Techno. 33, 593–602. 10.1111/1467-8535.00294

[B16] Cole D. A. McPherson A. E. (1993). Relation of family subsystems to adolescent depression: implementing a new family assessment strategy. J. Fam. Psychol. 7, 119–133. 10.1037/0893-3200.7.1.119

[B17] Creveling C. C. Varela R. E. Weems C. F. Corey D. M. (2010). Maternal control, cognitive style, and childhood anxiety: a test of a theoretical model in a multi-ethnic sample. J. Fam. Psychol. 24, 439–448. 10.1037/a0020388 20731490

[B18] Davidov E. Meuleman B. Cieciuch J. Schmidt P. Billiet J. (2014). Measurement equivalence in cross-national research. Ann. Rev. Soc. 40, 55–75. 10.1146/annurev-soc-071913-043137

[B19] Delsing M. J. Oud J. H. De Bruyn E. E. Van Aken M. A. (2003). Current and recollected perceptions of family relationships: the Social Relations Model approach applied to members of three generations. J. Fam. Psychol. 17, 445–459. 10.1037/0893-3200.17.4.445 14640796

[B20] Delsing M. J. Oud J. H. L. De Bruyn E. E. J. (2005a). Assessment of bidirectional influences between family relationships and adolescent problem behaviour. Eur. J. Psychol. Assess. 21, 226–231. 10.1027/1015-5759.21.4.226

[B21] Delsing M. J. N. H. Van Aken M. A. G. Oud J. H. L. De Bruyn E. E. J. Scholte R. H. J. (2005b). Family loyalty and adolescent problem behavior: the validity of the family group effect. J. Res. Adolesc. 15, 127–150. 10.1111/j.1532-7795.2005.00089.x

[B22] Dette-Hagenmeyer D. E. Erzinger A. B. Reichle B. (2014). The changing role of the father in the family. Eur. J. Dev. Psychol. 11, 129–135. 10.1080/17405629.2014.883313

[B23] Dunn J. Davies L. C. O’Connor T. G. Sturgess W. (2001). Family lives and friendships: the perspectives of children in step-, single-parent, and nonstep families. J. Fam. Psychol. 15, 272–287. 10.1037/0893-3200.15.2.272 11458633

[B24] Fitriana E. (2011). Confirmatory Factor Analysis of the Bandung Family Relation Test: A Simulation Study Comparing ML, DWLS, and WLS Estimation. Ph.D. thesis, Radboud University Nijmegen, Nijmegen.

[B25] Furman W. Buhrmester D. (1985). Children’s perceptions of the personal relationships in their social networks. Dev. Psychol. 21, 1016–1024.

[B26] Green J. M. Stanley C. Smith V. Goldwyn R. (2000). A new method of evaluating attachment representations on young school age children—the Manchester Child Attachment Story Task. Attach. Hum. Dev. 2, 42–64. 10.1080/146167300361318 11707892

[B27] Goodman R. (1997). The strengths and difficulties questionnaire. J. Child Psychol. Psychiatry 38, 581–586. 10.1111/j.1469-7610.1997.tb01545.x 9255702

[B28] Gurwitz S. B. Dodge K. A. (1975). Adults’ evaluations of a child as a function of sex of adult and sex of child. J. Pers. Soc. Psychol. 32, 822–828. 10.1037/0022-3514.32.5.822 1185515

[B29] Hambleton R. K. (2005). “Issues, Designs and Technical Guidelines for Adapting Tests Into Multiple Languages and Cultures,” in Adapting Psychological and Educational Tests for Cross-Cultural Assessment, eds Hambleton R. K. Merenda P. F. Spielberger C. D., NJ: Lawrence Erlbaum.

[B30] Hau K. T. Marsh H. W. (2004). The use of item parcels in structural equation modelling: non-normal data and small sample sizes. Br. J. Math. Stat. Psychol. 57, 327–351. 10.1111/j.2044-8317.2004.tb00142.x 15511312

[B31] Hooghe A. De Mol J. Baetens I. Zech E. (2013). The measurement of couple and family interactions and relationship quality in bereavement research. Fam. Sci. 4, 66–78. 10.1080/19424620.2013.821761

[B32] Hu L. Bentler P. M. (1999). Cutoff criteria for fit indexes in covariance structure analysis: conventional criteria versus new alternatives. Struct. Equ. Modeling 6, 1–55. 10.1080/10705519909540118

[B33] IBM Corp. (2012). IBM SPSS Statistics for Windows, Version 21.0. Armonk, NY: IBM Corp.

[B34] Kim P. I. Garber J. Ciesla J. A. Ellis B. J. (2008). Convergence among multiple methods of measuring positivity and negativity in the family environment: relation to depression in mothers and their children. J. Fam. Psychol. 22, 123–134. 10.1037/0893-3200.22.1.123 18266539 PMC2881156

[B35] Khorramdel L. Frebort M. (2011). Context effects on test performance: what about test order? Eur. J. Psychol. Assess. 27, 103–110. 10.1027/1015-5759/a000050

[B36] Mathijssen J. J. Koot H. M. Verhulst F. C. de Bruyn E. E. J. Oud J. H. L. (1998). The relationship between mutual family relations and child psychopathology. J. Child Psychol. Psychiatry 39, 477–487. 9599776

[B37] McDonald R. P. (1999). Test Theory: A Unified Treatment. Mahwah, NJ: L. Erlbaum Associates.

[B38] Milkie M. A. Simon R. W. Powell B. (1997). Through the eyes of children. Youths’ perceptions and evaluations of maternal and paternal roles. Soc. Psychol. Q. 60, 218–237.

[B39] Oud J. H. L. Welzen K. (1989). De Nijmeegse Gezinsrelatie Test: kinderversie [The Nijmegen Family Relations Test: Child version]. Lisse: Swets & Zeitlinger.

[B40] Parkin A. (2001). The Bene-Anthony Family Relations Test revisited: directions in the assessment of children’s perceptions of family relations. Br. J. Med. Psychol. 74, 323–349. 10.1348/000711201161019 11589325

[B41] Poehlmann J. Burnson C. Weymouth L. A. (2014). Early parenting, represented family relationships, and externalizing behavior problems in children born preterm. Attach. Hum. Dev. 16, 271–91. 10.1080/14616734.2014.884610 24580068 PMC4695717

[B42] Sim G. Horton M. (2005). “Performance and attitude of children in computer based versus paper based testing,” in Proceedings of World Conference on Educational Multimedia, Hypermedia and Telecommunications 2005, eds Kommers P. Richards G. (Chesapeake, VA: AACE), 3610–3614.

[B43] Stierlin H. (1978). Delegation und Familie [Delegation and family]. Frankfurt: Suhrkamp Verlag.

[B44] Strachan A. M. Lund M. E. Garcia J. A. (2010). Assessing children’s perceptions of family relationships: an interactive instrument for use in custody disputes. J. Child Custody 7, 192–216. 10.1080/15379418.2010.512236

[B45] Titze K. Schenck S. Zulauf-Logoz M. Lehmkuhl U. (2014). Assessing the quality of the parent–child relationship: validity and reliability of the Child–Parent Relationship Test (Chip-C). J. Child Fam. Stud. 23, 917–933. 10.1007/s10826-013-9749-7

[B46] Treiblmaier H. Filzmoser P. (2011). Benefits from using continuous rating scales in online survey research Paper Presented at the International Conference on Information Systems (ICIS), Shanghai, China.

[B47] Tynkkynen L. Vuori J. Salmela-Aro K. (2012). The role of psychological control, socioeconomic status and academic achievement in parents’ educational aspirations for their adolescent children. Eur. J. Dev. Psychol. 9, 695–710. 10.1080/17405629.2012.671581

[B48] Vandenberg R. J. Lance C. E. (2000). A review and synthesis of the measurement invariance literature: suggestions, practices, and recommendations for organizational research. Organ. Res. Methods 3, 4–69. 10.1177/109442810031002

